# Mesothelial cell inclusions mimicking adenocarcinoma in cervical lymph nodes in association with chylous effusion

**DOI:** 10.4103/0971-5851.71658

**Published:** 2010

**Authors:** Manu Goyal, Suseela Kodandapani, S. Nirni Sharanabasappa, Satya Dattatreya Palanki

**Affiliations:** *Department of Laboratory Medicine, Indo-American Cancer Institute and Research Centre, Hyderabad, India*; 1 *Department of Medical Oncology, Indo-American Cancer Institute and Research Centre, Hyderabad, India*

**Keywords:** *Adenocarcinoma*, *chylous effusion*, *epithelial membrane antigen*, *mesothelial cell inclusions*

## Abstract

Mesothelial cell inclusions in lymph nodes are of rare occurrence and can be mistaken as metastatic adenocarcinomas, mesothelioma or sinus histiocytosis. These are usually found in mediastinal and abdominal lymph nodes and are associated with effusions. We report a case of benign mesothelial cell inclusions in cervical lymph nodes, which was associated with chylous effusion, and immunohistochemistry revealed unusual weak cytoplasmic epithelial membrane antigen positivity in the cells.

## INTRODUCTION

Benign inclusions in lymph nodes are broadly classified as epithelial, nevomelanocytic and decidual.[1] Mesothelial cell inclusions, a type of epithelial inclusions, are of very rare occurrence, which could morphologically mimic as metastatic adenocarcinomas, mesothelioma or sinus histiocytosis.[1–5] These are usually found in mediastinal and abdominal lymph nodes and are associated with effusions.[1,6] These depict a very characteristic immunohistochemical pattern, which helps to distinguish these from the other entities. We report a case of benign mesothelial cell inclusions in cervical lymph nodes, which was associated with chylous effusion, and immunohistochemistry revealed an unusual weak cytoplasmic epithelial membrane antigen (EMA) positivity in the cells.

## CASE REPORT

A-16-year-old boy had presented with severe breathlessness, which was distressing even at rest. He had low grade fever and had a remitting and relapsing course. There was facial puffiness, and significant pedal edema. Chest radiograph revealed bilateral pleural effusion. Ultrasound examination of the abdomen revealed gross ascites; however, there was no organomegaly or any lymphadenopathy. Computerized tomography of the chest revealed pleuropericardial effusion with enlarged mediastinal, left supraclavicular and cervical lymph nodes, suggestive of lymphoma. His complete blood counts were: hemoglobin 15.2 g/dL; total leukocyte count 5.5×10^9^/L; platelets 2×10^9^/L. Peripheral smear was normal. Serum lactate dehydrogenase (LDH) level was 360 IU/L (<380 IU/L). Alpha-fetoprotein (AFP), beta-human chorionic gonadotropin (β-HCG) and carcinoembryonic antigen (CEA) levels screened in the serum were within normal limits.

Bone marrow evaluation was within normal limits. Both the ascitic and pleural fluids tapped were grossly milky white and sent for biochemical and cytological evaluation. Biochemical evaluation established the chylous nature of the fluid due to the presence of chylomicrons, markedly elevated triglycerides and normal cholesterol levels. Cytological evaluation revealed pleocytosis with the presence of benign lymphocytes and mesothelial cells only and was negative for malignancy.

**Figure 1:**
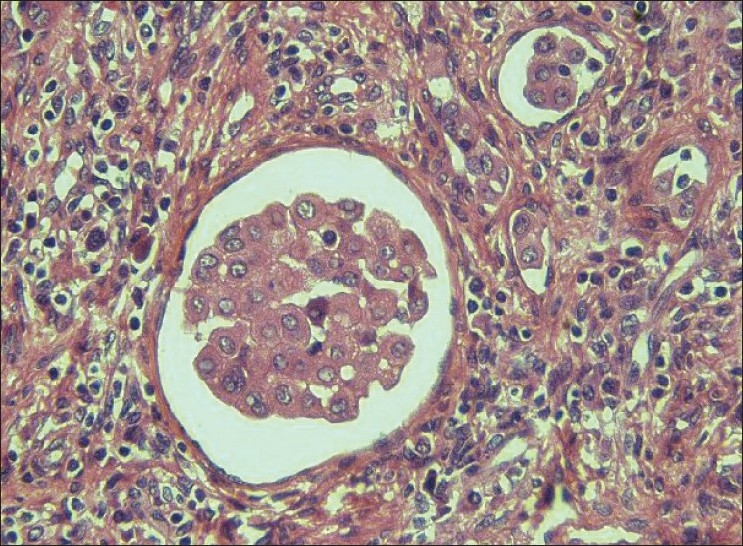
Photomicrograph of the lymph node shows clusters of polygonal cells in the sinuses. The nucleus is vesicular with small nucleolus and the nuclear–cytoplasmic ratio is low. There are tiny spaces seen in between the cells, mesothelial windows (hematoxylin and eosin stain, ×400)

**Figure 2:**
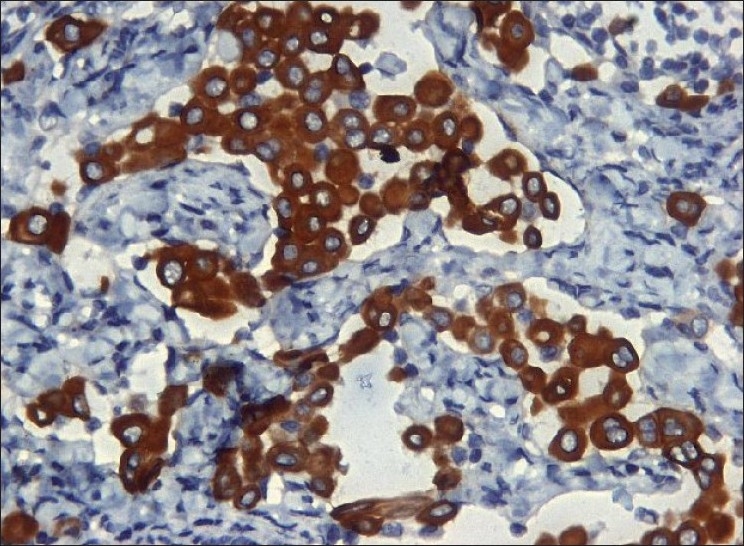
The cells show a strong cytoplasmic CK7 positivity (immunohistochemical stain, ×400)

**Figure 3:**
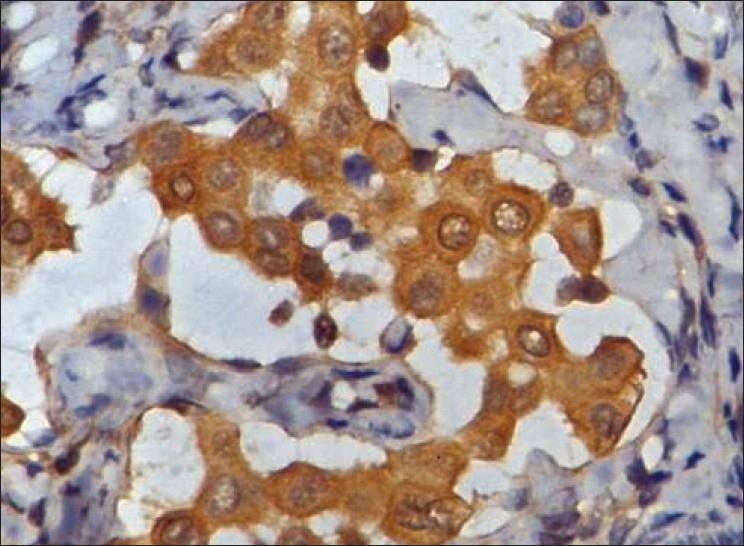
The polygonal cells show moderate nuclear and cytoplasmic calretinin positivity immunohistochemical stain, ×400)

Left cervical level V lymph node was biopsied for conclusive opinion. Grossly, there were four nodes ranging from 1 cm in diameter to 2×1.5×1 cm. Cut section of all nodes revealed yellowish tan to gray white, smooth to mottled surface. Microscopically, there were small clusters and singly scattered, round to polygonal cells, seen in the subcapsular and interfollicular sinuses of the nodes [Figure 1]. These cells had a round, vesicular nucleus with small nucleolus. The nuclear–cytoplasmic ratio was low. No mitotic activity was detected. There was no extranodal or parenchymal infiltration of the cells. There were tiny spaces seen in between the cells of the clusters (mesothelial windows) [Figure 1]. Immunohistochemistry was performed, which showed strong cytoplasmic positivity for cytokeratin (AE1/AE3) (1:25, Biocare, Concord, CA, USA) and cytokeratin 7 (CK7) [RTU (ready-to-use), PDM097, Biocare] [Figure 2] in these cells. These cells were negative for cytokeratin 20 (CK20) (RTU, AM315-5M, Biogenex, San Ramon, CA, USA), CD68 (RTU, AM416-5M, Biogenex), carcino-embryonic antigen (CEA) (1:200, Biocare), and thyroid transcription factor 1 (TTF-1) (1:25, Biocare). Calretinin (RTU, AR413-5R, Biogenex) revealed nuclear and cytoplasmic immunoreactivity in these cells [Figure 3] and EMA (RTU, N1504, DAKO, Carpinteria, CA, USA) staining was very weak and cytoplasmic. CD34 (RTU, N1632, DAKO) highlighted the endothelial cell lining and the presence of cells within the sinusoids. Ki-67 (1:100, Biocare) staining did not show any proliferation in the epithelial cells. CD20 (RTU, N1502, DAKO) and CD3 (1:200, Biocare) revealed normal lymph nodal architecture. No lymphoproliferative disorder was detected in the nodes examined.

The breathlessness of the patient was progressive and the patient collapsed due to cardiorespiratory arrest.

## DISCUSSION

Benign inclusions in lymph nodes are of various types. These are uncommon in occurrence and, thus, mistaken as metastatic carcinoma or melanomas depending on the type of inclusions.[1,6] The different types of inclusions are associated with a preferential topographic location of the lymph nodes, e.g., breast tissue in the axilla, thyroid follicles in the cervical, nevus cells in the axilla or inguinal regions.[6] However, there are reports of inclusions in unusual sites, such as the nevus cells in the cervical nodes.[7]

Mesothelial cell inclusions are of very rare occurrence and usually involve the mediastinal and retroperitoneal groups of lymph nodes.[1–4,6] The involvement of cervical nodes is unknown to the best of our knowledge. These are usually associated with hyperplasia and inflammation of the associated serosal membranes.[1,4,6] Our case is unusual as it was associated with chylous effusion and the inclusions were found in cervical nodes. The clinical significance of the inclusions associated with chylous effusion is still not known and needs to be established, if any.

The main problem with mesothelial inclusions is that these mimic sinus histiocytosis, metastatic adenocarcinoma, and metastatic mesothelioma.[1–3,5] The morphology of the cells, cytokeratin positivity and CD68 negativity confirm the epithelial nature of the cells and not histiocytes. The distinction from metastasis from benign inclusions is much more challenging. The reasons in favor of benign mesothelial cells are the bland nature of the cells, mesothelial windows, absence of mitosis and lack of parenchymal infiltration, highlighted by the CD34 staining pattern.[1,5] Their characteristic immunoprofile showing positivity for AE1/AE3, CK7, calretinin and negativity for CK20, TTF-1, and CEA also establish their mesothelial origin.[5] Ki-67 immunostaining did not reveal any proliferating cells. EMA immunopositivity is seen in only 3–4% of reactive mesothelial cells as compared to malignant mesothelioma.[8,9] However, the malignant mesothelial cells are characterized by strong membranous reactivity.[8,9] The present case showed weak cytoplasmic EMA positivity. We consider the cells as benign based on the above features and also a thorough clinical, radiological and biochemical evaluation was negative for any malignant disease. Even the cytological evaluation of the pleural and ascitic fluids did not contain malignant cells.

The mechanism of the mesothelial cell inclusions is also distinct as compared to other inclusions.[5] Developmental or metaplastic theories as in glandular inclusions cannot be applied here due to intrasinusoidal location.[1,5] The postulated mechanism is the transportation of these cells through the lymphatics to the lymph node during injury or manipulation at the primary site of the origin.[1,5,10] As compared to malignant cells, which are capable of proliferation, these cells are not usually found in lymph nodes, as they undergo a degeneration process, and thus it becomes difficult to find them.[1,6] Possibly, all these are responsible for the presence of the inclusions in the sinuses and not in the parenchyma.

Although the cause of death could not be established in our patient, we presume that some other undiagnosed pathology resulted in these mesothelial inclusions, which were an incidental finding. It is important to note that the presence of these benign and innocuous epithelial elements in the sinuses may lead to a misdiagnosis of metastatic carcinoma to the unwary.

## CONCLUSION

Mesothelial cell inclusions in lymph nodes are a rare occurrence and may be mistaken for malignant adenocarcinoma or mesothelioma. The cellular characteristics with the immunoprofile should be evaluated carefully to prevent such errors, even though these occur in rare sites.
